# Dynamic Regulation of HIF1α and Oxygen-Sensing Factors in Cyclic Bovine Corpus Luteum and During LPS Challenge

**DOI:** 10.3390/ani15040595

**Published:** 2025-02-19

**Authors:** Luiz Antonio Berto Gomes, Olivia Eilers Smith, Heinrich Bollwein, Mariusz Pawel Kowalewski

**Affiliations:** 1 Institute of Veterinary Anatomy, Vetsuisse Faculty, University of Zurich, CH-8057 Zurich, Switzerland; luizantonio.bertogomes@uzh.ch (L.A.B.G.); olivia.smith@uzh.ch (O.E.S.); 2 Clinic of Reproductive Medicine, Vetsuisse Faculty, University of Zurich, CH-8057 Zurich, Switzerland; heinrich.bollwein@uzh.ch; 3 AgroVet-Strickhof, Vetsuisse Faculty, University of Zurich, CH-8315 Eschikon, Switzerland; 4 Center for Clinical Studies (ZKS), Vetsuisse Faculty, University of Zurich, CH-8057 Zurich, Switzerland

**Keywords:** bovine corpus luteum, hypoxia, HIF1α, LPS

## Abstract

Understanding the corpus luteum (CL) and its role in cattle reproduction is crucial, particularly as it is a progesterone source for the establishment and maintenance of pregnancy. Reduced oxygen levels significantly impact these processes. This study investigated the effects of the luteal stage on the spatio-temporal gene expression patterns of HIF1α and oxygen-sensing factors, as well as the impact of lipopolysaccharide (LPS)-induced inflammation on these factors. Endothelial inflammatory responses were also addressed. The samples included CL collected at the early, mid, and late stages, as well as biopsies from mid-luteal stage cows treated either with saline (controls) or LPS. Samples collected in subsequent cycles assessed potential carryover effects. RT-PCR revealed upregulation of HIF1α, PHD1, PHD3, FIH, and VHL encoding genes in the mid-luteal stage. In situ hybridization revealed the compartmentalization of HIF1α and its regulators within the luteal and endothelial cells, suggesting their cell-specific roles. LPS treatment affected *PHD1* and *PHD3* expression, while increasing endothelial pro-inflammatory factors *ICAM1* and *NFκB*, suggesting vascular inflammation and modulated oxygen sensing. These findings reveal new insights into the spatio-temporal expression of HIF1α-regulating factors in the CL, highlighting their potential role in controlling luteal function, detailing their cellular compartmentalization, and the effects of LPS-mediated inflammatory responses.

## Simple Summary

The corpus luteum is a temporary gland that provides progesterone necessary for the establishment and maintenance of pregnancy. Its function is influenced by changes in oxygen concentrations and can be affected by inflammation, including from bacterial infections. This study evaluated how HIF1α and oxygen-sensing factors are expressed and regulated in the bovine corpus luteum at different luteal stages. It also assessed the impact of bacterial LPS endotoxin on their expression, as well as on the expression of selected endothelial pro-inflammatory markers. These findings widen our understanding of how these factors influence the luteal phase and how they respond to LPS-mediated inflammation.

## 1. Introduction

Acting as a transient gland and primary source of progesterone (P4), the corpus luteum (CL) is an essential player in bovine reproductive physiology. In the cyclic cow, the CL lifespan ranges from 14 to 18 days when there is no pregnancy involved, with the luteal phase being divided into three developmental stages, i.e., the early, mid-, and late luteal stages [1,2]. The origin of the CL is in the preovulatory follicles, the vascularization of which is limited to the theca cell layer, while the granulosa cells, which produce oestrogens, function under lower oxygen tension, referred to as hypoxia [1,2,3]. Following ovulation, the remaining granulosa and theca interna cells undergo luteinization, becoming large and small luteal cells, respectively, and shifting their activity to P4 production [1,2,3]. Hypoxia-inducible factor 1 alpha (HIF1α) is one of the most prominent regulators of cellular responses to hypoxia [4]. As a part of heterodimer HIF1 complexes, HIF1α acts predominantly as an oxygen-dependent transcriptional activator [4]. The luteinization process is accompanied by active angiogenesis and, as the CL forms under physiologically low oxygen content, the elevated expression of HIF1α induces processes such as steroidogenesis, including direct transcriptional regulation of steroidogenic acute regulatory protein (STAR) expression, glucose uptake, and autophagy [5,6,7]. During the regression of the CL, luteal blood flow decreases, due in large part to vascular occlusion, promoting a local hypoxic environment, which, in turn, leads to a reduction in both P4 secretion and the size of the CL [8,9,10]. In this context, lower oxygen tension generates reactive oxygen species (ROS), damages the mitochondria, and inhibits the activity of P450scc, an important enzyme for the conversion of cholesterol into pregnenolone [11,12]. Thus, depending on the stage of luteal development, HIF-complexes, and particularly HIF1α, appear to exert both positive and negative effects on steroidogenic cells. Accordingly, a biphasic pattern of HIF1α activity upon STAR expression was recently implied by observations made in a granulosa cell model [13]. Given the interplay between lower oxygen tension and HIF1α in regulating CL lifespan and function, a better understanding of the underlying regulatory mechanisms is required.

HIF1α is ubiquitously expressed in all tissues [14]. For its transcriptional activation, it dimerizes with HIF1β/ aryl hydrocarbon receptor nuclear translocator (ARNT) through the helix-loop-helix (bHLH)-Per-Arnt-Sim domain and then binds to the hypoxia response element (HRE) of target genes [15]. Besides its oxygen-dependent activation, the expression of HIF1α can be increased through follicle-stimulating hormone (FSH) or insulin-like growth factor 1 (IGF1) in an oxygen-independent manner [16,17], while ARNT is constitutively expressed [18,19]. The availability of HIF1α is regulated enzymatically by oxygen sensing factors, namely factor inhibiting HIF (FIH; encoded by *HIF1AN*) and prolyl-hydroxylases (PHDs). Three isoforms of PHDs are known: PHD1 (encoded by *EGLN2*), PHD2 (encoded by *EGLN1*), and PHD3 (encoded by *EGLN3*) [20,21]. While FIH acts by hydroxylating the asparagine residue in the C-terminal transactivation domain of HIF1α, thereby preventing the interaction of HIF with the transcriptional co-activator CBP/P300 [22,23,24], PHDs hydroxylate specific proline residues on HIF1α, allowing its recognition by the von Hippel–Lindau (VHL) protein, an E3 ubiquitin ligase complex component [25]. Once recognized by VHL, HIF1α is ubiquitinated and targeted for proteasomal degradation [26]. While the regulatory pathways of HIF1α have been thoroughly explored, much less is known about regulation of its availability in the CL.

The susceptibility of the CL to external challenges, such as pathogen infections affecting reproductive organs, adds another layer of complexity to the study of the bovine CL. Uterine and mammary diseases associated with bacterial infections, including some forms of metritis and mastitis, respectively, have been shown to play a role in the breeding efficiency of dairy cows [27,28]. Gram-negative bacteria like Escherichia coli are known to play an important role in bovine infertility and subfertility, primarily due to their systemic inflammatory response triggered by the lipopolysaccharide (LPS) endotoxin [29,30,31]. LPS has been shown to impact CL function, luteinizing hormone (LH) surge, and steroidogenesis [32,33]. In vitro studies have shown that LPS affects ovine endothelial luteal cells by disrupting gap junctional communication and altering tube formation, and in porcine granulosa cells, LPS reduces P4 concentration [34,35,36]. Less is known about the effects of LPS on the bovine CL, and the possible role it may have in regulating HIF1α. Additionally, in vitro, LPS has been shown to induce inflammatory processes in ovine luteal endothelial cells, as well as in bovine and mouse endothelial cells [35,37,38], associated with the upregulated expression of proinflammatory factors such as intercellular adhesion molecule 1 (ICAM1) and nuclear factor kappa B (NFκB) [35,37,39,40].

The potential relationship between lower oxygen tension, HIF1α, and the response of the bovine CL to LPS remains unknown. Here, it is hypothesized that HIF1α regulators could exert significant influence over CL development via dynamic spatio-temporal expression patterns, and that the treatment of cyclic cows with LPS could affect the expression of HIF1α and its regulators. Therefore, the expression of HIF1α and its oxygen sensing regulatory factors in the bovine CL lifespan was evaluated, and their localization was determined. Additionally, we evaluated whether LPS can affect luteal function via the regulation of these factors, and whether proinflammation cytokines, ICAM1 and NFκB, could be involved in the LPS-mediated effects in the CL of treated cows.

## 2. Materials and Methods

### 2.1. Tissue Material

All tissue material used in the present study was derived from previous experiments [32,33]. Samples were either stored at −80 °C or available as formalin-fixed paraffin-embedded tissue (FFPE) blocks.

#### 2.1.1. CL Samples Collected at Different Stages of Luteal Development

*Study 1:* Ovaries were collected between March and April from 14 clinically healthy, non-lactating Holstein cows with an age between 2.5 and 8.3 years and a body condition score (BCS) between 2.75 and 3.5. The cows were housed in a tie stall barn and fed high-quality hay, grass, concentrates/minerals, and had ad libitum access to water. They were slaughtered at a commercial slaughterhouse. The experimental procedures followed the Swiss Federal Law on Animal Protection and were approved by the Committee of Animal Experiments of the Canton Fribourg, Switzerland (application 25076, from Lüttgenau et al., 2016 [32]). Ovulation (Day 1) was synchronized using a modified ovulation synchronization (Ovsynch) protocol after normal cycle activity was confirmed in each cow by ultrasonography. Briefly, cows received 10 μg of GnRH analogue buserelin (Receptal; MSD Animal Health GmbH, Luzern, Switzerland) via intramuscular injection (i.m.). After seven days, 15 mg of PGF2α analogue luprostiol i.m. (Prosolvin; Virbac AG, Glattbrugg, Switzerland) was administered, followed by a second 10 μg of buserelin i.m. 60 h after the PGF2α analogue. All cows ovulated within 36 h of the final injection. Beginning 17 days post-ovulation, transrectal ultrasonography was conducted every 2 days to detect the subsequent ovulation (Day 1). Following this, examinations were performed every 2–3 days to track the normal development of the CL. The final examination was carried out within 6 h prior to the cows being slaughtered, which took place at different stages of luteal development [32]. CLs were categorized according to [1,41], based on days post-ovulation, accordingly: early (5–7 days post-ovulation; n = 4), mid- (8–12 days post-ovulation; n = 5), and late (13–18 days post-ovulation, n = 5) luteal stage. CL samples were collected and processed immediately after slaughter, incubated in RNA later (Ambion Biotechnology GmbH, Wiesbaden, Germany) for 24 h at 4 °C, and stored at −80 °C for further analysis. For in situ hybridization (ISH), tissue samples were fixed in 10% neutral phosphate-buffered formalin and finally processed for embedding in paraffin-equivalent Histo-Comp (Vogel Medizintechnik, Giessen, Germany).

#### 2.1.2. CL Biopsies from Control and LPS-Treated Cows

*Study 2:* Control and LPS-treated luteal biopsies (from Herzog et al., 2012 [33]; Lower Saxony Federal State Office for Consumer Protection and Food Safety, 33.9-42502—04-09/1782, in accordance with German legislation on animal welfare) were used and collected from seven (n = 7) clinically healthy primi- and pluriparous non-lactating Holstein cows (*Bos taurus*) with an age between 2.4 and 8.2 years and a BCS ranging from 2.75 to 3.25. The cows were housed in individual boxes and fed ad libitum with hay and water and were submitted in March 2010 to transrectal ultrasonography at 12, 24 and 36 h after the modified Ovsynch protocol to detect the time of ovulation (Day 1 of oestrous cycle). Cows were first treated to collect control samples: on Day 10 of the first oestrous cycle, cows received 10 mL 0.9% NaCl intravenously over 1 min. CL sample tissue was collected 12 h after this treatment (Control—1st cycle) and collected on day 10 of the subsequent cycle (Control—2nd cycle). Cows then went through the LPS-treated cycles. To ensure consistency, they received the Ovsynch protocol and were treated with *E. coli* LPS (O55:B5, Sigma-Aldrich, Buchs, Switzerland, 0.5 μg/kg body weight diluted in 10 mL sterile water) intravenously over 1 min on day 10. CL tissue samples were collected 12 h after the LPS treatment (LPS—1st cycle) and once again on day 10 of the subsequent cycle (LPS—2nd cycle) to evaluate systemic carryover effects. Biopsies were immediately processed and stored at −80 °C for further analysis. Serum P4 concentrations were measured prior to the NaCl or LPS administration and are reported in [33]. The concentration of LPS followed previous reports in which it was demonstrated to affect luteal function and morphology and trigger a systemic response without risking the life of the animals [33].

### 2.2. RNA Isolation, Reverse Transcription, and Semi-Quantitative Real-Time TaqMan PCR

Total RNA was isolated from all samples using TRIzol reagent (Invitrogen, Carlsbad, CA, USA) according to the manufacturer’s protocol. A NanoDrop 2000 Spectrophotometer (Thermo Fisher Scientific AG, Reinach, Switzerland) was used for quantification and quality assessment of the isolated total RNA. Possible genomic DNA contamination was removed with RQ1 RNase-free DNase treatment (Promega, Duebendorf, Switzerland). For the CL samples derived from the different stages of luteal development, complementary DNA (cDNA) was synthesized with the MultiScribe Reverse Transcriptase, using random hexamers as primers (Applied Biosystems, Thermo Fisher, Waltham, MA, USA). For biopsies, due to the low tissue input, the High-Capacity cDNA Reverse Transcription Kit (Applied Biosystems, Thermo Fisher, Waltham, MA, USA) was used for Reverse Transcription. Semi-quantitative real-time TaqMan PCR was performed in duplicate in a 96-well optical plate with the FastStart Universal Probe Master (Roche Diagnostics AG, Rotkreuz, Switzerland) in an automated ABI PRISM 7500 Sequence Detection System fluorometer (Applied Biosystems, Thermo Fisher, Waltham, MA, USA). Commercially available TaqMan systems were obtained from Applied Biosystems by Thermo Fisher (Waltham, MA, USA). In-house-designed TaqMan systems, primers (based on published coding sequences), and 6-carboxyfluorescein (6-FAM)- and 6-carboxytetramethylrhodamine (TAMRA)-labelled probes were ordered from Microsynth (Balgach, Switzerland). Table 1 details the primers and probes used.

Autoclaved water and non-reverse-transcribed RNA (RT-minus control) were used instead of cDNA as negative controls. The relative gene expression was evaluated using the ΔΔ Ct method, normalized to the expression of the reference genes. *GAPDH* and *BACTIN* were used initially, and their stability was analysed using the online tool RefFinder [42]. Consequently, *BACTIN* was excluded from the analysis of cyclic CLs.

### 2.3. In Situ Hybridization (ISH)

HIF1α and the factors involved in its stabilization, PHD1, -2, -3, VHL, and FIH, were localized at the mRNA level with non-radioactive in situ hybridization (ISH), as previously described [43].

Complementary RNA (cRNA) probes were generated from cDNA products amplified via PCR using bovine-specific primers (refer to Table 2; obtained from Microsynth). Electrophoresis was carried out on a 2% agarose gel stained with ethidium bromide to separate the PCR products, which were purified with QIAquick Gel Extraction Kit (Qiagen GmBH, Hilden, Germany), followed by subcloning into pGEM-T plasmid (Promega, Dubendorf, Switzerland), and bacteria transformation (XL-1Blue Competent Cells; Agilent, Waldbronn, Germany) for blue/white selection. The PureYield^TM^ Plasmid Miniprep System (Promega) was used to isolate monoclonal colonies carrying the insert. The colonies were submitted to digestion with NotI and NcoI restriction enzymes and were subsequently confirmed by Sanger sequencing (Microsynth) to determine the sense and antisense direction of the products. Following that, sense and antisense digoxigenin (DIG)-labeled cRNA probes were synthesized using the DIG-RNA labelling kit (Roche Diagnostics AG, Basel, Switzerland) according to the manufacturer’s protocol. The efficiency of the DIG-labelled riboprobes was assessed by dot blot on positively charged nylon membranes through serial dilutions stained with 5-bromo-4-chloro-3-indolyl phosphate (BCIP)/nitroblue tetrazolium (NBT) colour development substrate (Roche Diagnostics, Basel, Switzerland). Non-radioactive ISH was performed on paraffin-embedded CL cut into 2 μm thick slices. After the sections were dewaxed with xylol and rehydrated with a series of ethanol incubations, the sections were digested with proteinase K (70 μg/mL; Sigma-Aldrich, Buchs, Switzerland) for 25 min at 37 °C, and post-fixed with 4% paraformaldehyde [43].

Overnight hybridization took place in a formamide chamber at 4 °C. Dig-labelled cRNA was detected by incubating the tissue with serum-free alkaline phosphatase (AP)-conjugated sheep anti-DIG Fab fragment antibody (diluted 1:5000; Roche Diagnostics, Basel, Switzerland) overnight at 4 °C. Prior to the incubation with the antibody, tissue samples were incubated for one hour with 3% goat serum. Additionally, levamisole was used to inhibit endogenous alkaline phosphatases. The conjugated signals were visualized using BCIP/NBT colour development substrate (Roche Diagnostics, Basel, Switzerland), and the slides were mounted with phenol-free Kaiser’s glycerol gelatine. At least three slides were processed and observed for each cRNA probe set per cycle stage; representative pictures are shown.

### 2.4. Statistical Analysis

Statistical analyses for relative gene expression were performed using the GraphPad 3.06 software (San Diego, CA, USA). When data were normally distributed, a one-way analysis of variance (ANOVA) was applied, followed by the Tukey–Kramer test. For non-normally distributed data, the Kruskal–Wallis test (non-parametric ANOVA) followed by Dunn’s test, was performed. For all procedures, *p* ≤ 0.05 was considered statistically significant. The relative gene expression was presented as the mean ± standard error of the mean (SEM).

## 3. Results

### 3.1. HIF1α and Its Regulatory Factors During the Bovine Luteal Lifespan

**Figure 1:**
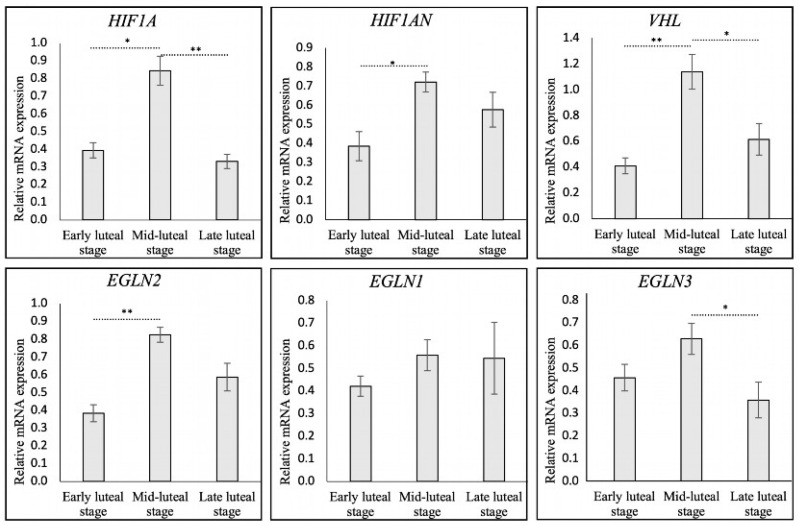
Relative mRNA expression of *HIF1A* (HIF1α) and HIF1α-regulating factors, i.e., *EGLN2* (PHD1), *EGLN1* (PHD2), *EGLN3* (PHD3), *HIF1AN* (FIH), and *VHL* in bovine CL during early, mid-, and late luteal stages. Relative gene expression as determined by semi-quantitative real-time (TaqMan) PCR is presented as mean ± standard error of mean (±SEM). Bars with asterisks differ at * *p* < 0.05 and ** *p* < 0.01.

To evaluate the gene expression levels of HIF1α and its regulators during the different stages of CL development, semi-quantitative TaqMan RT-PCR involving HIF1α (*HIF1A*), PHD1 (*EGLN2*), PHD2 (*EGLN1*), PHD3 (*EGLN3*), FIH (*HIF1AN*), and VHL (*VHL*) was performed. The passage of time significantly affected *EGLN3* (*p* > 0.05) and *VHL* (*p* = 0.001) (as evaluated by one-way ANOVA), as well as *HIF1A* (*p* < 0.001), *EGLN2* (*p* = 0.01), and *HIF1AN* (*p* < 0.05), while *EGLN1* remained unaffected (*p* > 0.05) (as evaluated by the Kruskal–Wallis test). These findings (Figure 1) highlighted dynamic fluctuations in gene expression patterns throughout the luteal lifespan. *HIF1A* expression was significantly upregulated (*p* < 0.05) during luteal development, followed by a marked downregulation in the late luteal stage (*p* < 0.01). In the mid-luteal stage, increased expression of *HIF1AN*, *EGLN2*, and *VHL* was observed (*p* < 0.05, *p* < 0.01, and *p* < 0.01, respectively; details in Figure 1). Subsequently, during the late luteal stage, the expression of *VHL* decreased significantly (*p* < 0.05), while *EGLN2* and *HIF1AN* levels remained stable (*p* > 0.05). Furthermore, the expression of *EGLN3* decreased (*p* < 0.05) during the late luteal stage. No significant changes were observed for transcripts encoding for *EGLN1* (*p* > 0.05).

### 3.2. In Situ Localization of HIF1α and Its Regulatory Factors in the Bovine CL

**Figure 2:**
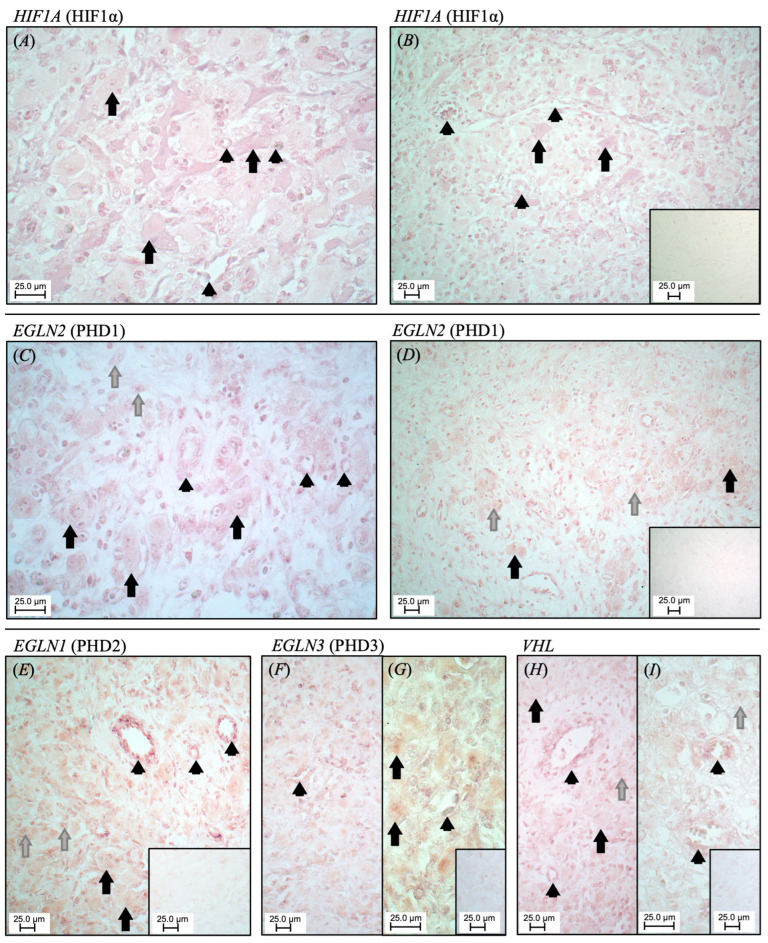
Luteal localization of transcripts (*mRNA*) encoding for *HIF1A* (HIF1α) (**A**,**B**), *EGLN2* (PHD1) (**C**,**D**), *EGLN1* (PHD2) (**E**), *EGLN3* (PHD3) (**F**,**G**), and *VHL* (**H**,**I**) in bovine CL, shown at mid-luteal stage. Black arrow: large luteal cells; grey arrow: small luteal cells; arrowhead: vessel. Explanations in text. The sense probes were employed as negative controls (smaller inserts at the same magnification to (**B**,**D**,**E**,**G**,**I**)).

Due to the lack of specificity of available antibodies, an attempt to investigate the protein expression patterns using Western Blot and immunohistochemistry failed. As an alternative method, we resorted to in situ hybridization (ISH) to localize the transcripts, thus *mRNA* encoding for HIF1α (*HIF1A*), PHD1 (*EGLN2*), PHD2 (*EGLN1*), PHD3 (*EGLN3*), and *VHL*. Positive staining against *HIF1A* (Figure 2A,B) was mainly detected in large luteal cells, and endothelial cells of capillary vessels, with additional signals in smaller vessels (e.g., arterioles, Figure 2B). *EGLN2* signals (Figure 2C,D) were mainly localized within the vessel, encompassing both the tunica intima and media, and were comparatively less abundant in luteal cells. The signals of *EGLN1* (Figure 2E) were strongly observed in vessels, particularly in the tunica intima and media, and mostly in larger vessels. Within luteal cells, *EGLN1* signals appeared to be ubiquitously distributed in both small and large cells, albeit exhibiting a weaker intensity compared to vessels. The signals of *EGLN3* (Figure 2F,G) and *VHL* (Figure 2H,I) appeared more pronounced in the tunica intima and media of the vessels than in luteal cells, displaying a distribution pattern similar to that observed for *ELGN1* signals. Sense probes were used for negative controls, and no staining was observed.

### 3.3. HIF1α and Its Regulatory Factors Following LPS Treatment

**Figure 3:**
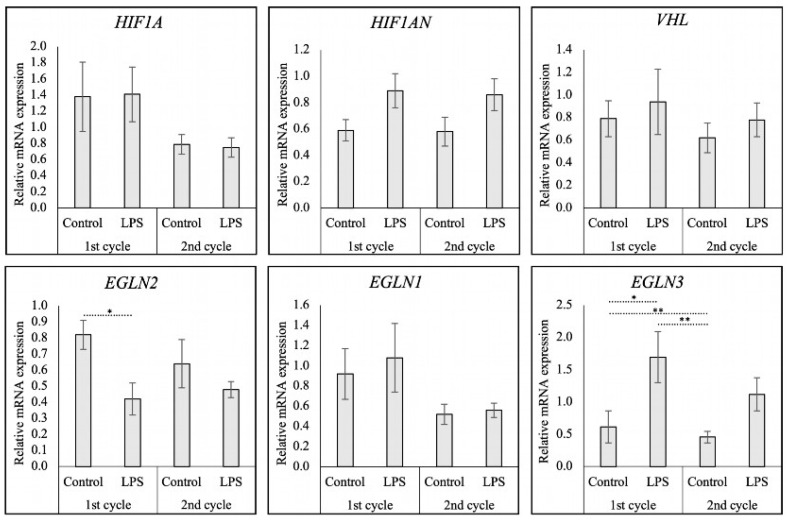
Relative mRNA expression of *HIF1A* (HIF1α) and its regulatory factors, *EGLN2* (PHD1), *EGLN1* (PHD2), *EGLN3* (PHD3), *HIF1AN* (FIH), and *VHL* during and after *E. coli*-LPS treatment. Relative gene expression as determined by semi-quantitative real-time (TaqMan) PCR is presented as mean ± standard error of mean (±SEM). Bars with asterisks differ at * *p* < 0.05 and ** *p* < 0.01.

LPS treatment had significant effects upon the expression of *EGLN2* (PHD1) (*p* < 0.05) and *EGLN3* (PHD3) (*p* < 0.001) compared to the controls (determined by Kruskal–Wallis test). Other factors, including *HIF1A* (*p* > 0.05), *EGLN1* (PHD2) (*p* > 0.05), *HIF1AN* (FIH) (*p* > 0.05), and *VHL* (*p* > 0.05), were unaffected by the treatment. As significant variations were observed in *EGLN2* and *EGLN3*, post-tests were conducted for these two factors, revealing that the administration of LPS (Figure 3) decreased the transcript levels of *EGLN2* compared with the control group in the first cycle (*p* < 0.05). No carryover effects were observed in subsequent cycles in either the LPS-treated or control groups. Conversely, the mRNA expression of *EGLN3* increased during LPS treatment compared to the control cycle (*p* < 0.05). A lower expression of *EGLN3* was observed in the second cycle control samples compared to the control from the first cycle (*p* < 0.01), possibly due to a cycle-dependent variation.

### 3.4. The Expression of ICAM1 and NFΚB2 in the CL of LPS-Treated Cows

**Figure 4:**
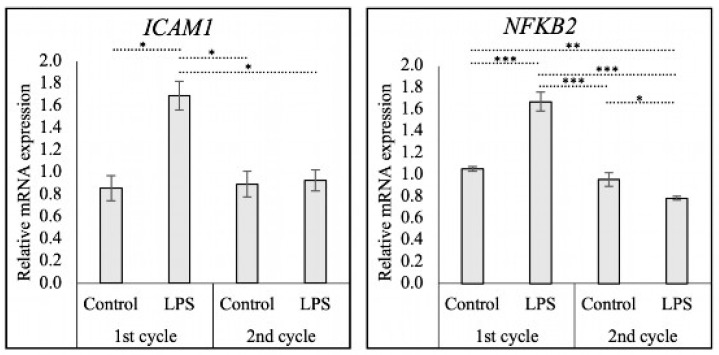
Relative mRNA expression of *ICAM1* and *NFKB2* during and after *E. coli*-LPS treatment. Relative gene expression as determined by semi-quantitative real-time (TaqMan) PCR is presented as mean ± standard error of mean (±SEM). Bars with asterisks differ at * *p* < 0.05, ** *p* < 0.01, and *** *p* < 0.001.

LPS treatment affected the luteal expression of ICAM1 (*p* < 0.01) and NFΚB2 (*p* < 0.001) (one-way ANOVA) (Figure 4). In the first cycle, both *ICAM1* (*p* < 0.05) and *NFKB2* (*p* < 0.001) mRNA levels were significantly higher in the LPS-treated samples than in the non-treated controls. In the second cycle, no difference in *ICAM1* mRNA levels was observed between the two groups (*p* > 0.05), while the expression of *NFKB2* was lower in the biopsies deriving from previously LPS-treated animals compared with controls (*p* < 0.05). Additionally, the expression of both *ICAM1* (*p* < 0.05) and *NFKB2* (*p* < 0.001) in LPS-treated animals was lower in the subsequent cycle when compared to the first cycle, while the expression in control animals remained constant. Interestingly, the expression of *NFKB2* in biopsies collected in the subsequent cycle of LPS-treated animals was lower than the expression in control biopsies in the first (*p* < 0.01) and second cycle (*p* < 0.05).

## 4. Discussion

The development of the bovine CL is characterized by reduced oxygen tension stimulating the vascularization of the rapidly developing gland [7,44]. This results in an increase in HIF1α expression, which stimulates steroidogenesis-related factors, angiogenesis, glucose uptake, and autophagy [5]. At the other end of the luteal life span, during luteal regression, a decrease in vascular supply leads to hypoxia, affecting P4 production, facilitating apoptosis, and contributing to the formation of the corpus albicans [5]. Thus, apparently, the effects evoked by HIF1α depend upon the functional status of the CL and its developmental stage, although there is only limited knowledge about factors regulating the availability of HIF1α during the luteal lifespan. Here, our data imply that, based on their spatio-temporal distribution, HIF1α regulators may exert a significant influence over the development and the function dynamics of the bovine CL.

In accordance with previous findings [45], we found that *HIF1α* mRNA levels reached their highest abundance at the mid-luteal stage, followed by a significant and progressive decline towards the luteal regression stage. This expression pattern of HIF1α appeared to be correlated with the increased transcriptional availability of *STAR* towards the mid-luteal stage [32]. Indeed, in support of the positive effects of HIF1α upon steroidogenic cell function, the direct transcriptional regulation of the *STAR* promoter by HIF1α has been shown in other models, e.g., in murine granulosa cells [6]. Moreover, reduced O_2_ tension showed positive effects upon STAR expression and steroidogenesis in both murine granulosa cells and bovine luteal cells [6,46], while functional blockage of HIF1α suppressed their steroidogenic activity [6,13,47]. Yet, no studies have considered the expression of HIF1α controlling factors. Thus, to our knowledge, our study is the first to evaluate the machinery regulating HIF1α at different stages of the developing CL.

The significantly increased expression of PHD1-, FIH-, and VHL-encoding transcripts during the mid-luteal stage suggests their combined action to regulate HIF1α activity and prevent its excessive accumulation while it is still needed for luteal development. This is in accordance with several previous studies, in which cobalt chloride (CoCl_2_) was used to block HIF1α degradation, resulting in aberrantly high HIF1α levels, and causing detrimental effects on the functionality of steroidogenic cells [48,49,50,51]. The observed decreased expression in PHD3 during the late luteal stage implies a potential shift in the regulatory mechanisms governing HIF1α regulation during CL regression. Apart from its interaction with HIF1α, PHD3 has also been suggested to hydroxylate other targets, e.g., those involved in the glucose metabolism pathway, regulating their availability, as shown in other models, e.g., human renal carcinoma cells and mice hepatocytes [52,53]. Glucose metabolism plays an important role in the function of the CL, being crucial for providing the energy substrate for ATP generation [54,55]. Notably, the bovine CL expresses key glucose transporter proteins, including Glut 1, -3, and -4 [56], and, additionally, GLUTs have been shown to be targets of HIF1α transcriptional activity in different species [49,56,57]. The bovine CL exhibits higher glucose uptake and metabolism during the early and mid-luteal stage, and glucose is required for the luteal production of P4 [55,57,58]. Consequently, our results may indicate the possible importance of PHD3 for the provision of glucose to the early and mid-luteal stage CL, in addition to its role in regulating different HIF1α-mediated functions.

In mouse granulosa cells, PHD2 was identified as a potential key regulator of HIF1α expression, more specifically in regulating STAR expression and steroidogenesis at lower O_2_ saturation [13]. Additionally, the stabilization of HIF1α by inhibiting PHDs increased STAR expression in a dose-dependent manner, and this effect was suppressed when HIF1α activity was inhibited [13]. When exaggeratedly stabilized by PHD blockage, the overabundance of HIF1α was associated with decreased STAR expression, fitting the results obtained from studies utilizing CoCl_2_ [51,59]. With that study, the biphasic involvement of HIF1α in regulating steroidogenic cell function was substantiated. Despite the absence of change at the transcriptional level observed in the present study for PHD2 during luteal development in cows, and considering its spatial cellular distribution within the CL (discussed below) and previous findings [13], PHD2 remains a factor deserving more attention in future studies focusing on the ovarian stabilization of HIF1α. Cumulatively, our findings regarding the differential expression patterns of PHDs shed light on the intricate regulation of HIF1α levels during the bovine CL lifespan. The dynamics in the expression of these regulators highlight the importance of precise, time-dependent HIF1α regulation for maintaining luteal function and suggest a nuanced interplay between oxygen-sensitive factors within the CL microenvironment.

Applying ISH for *mRNA* tissue localization, we were able to demonstrate a clear compartmentalization of the distribution of HIF1α and its regulating factors within the mid-luteal stage bovine CL, highlighting their spatial expression at the transcript level. These results are among the most important findings from the present study. First, in accordance with the findings of Berisha et al. (2017) [60], we found HIF1α-positive signals predominantly in lutein cells, with a particularly strong abundance of transcripts in the large luteal cells. It needs to be emphasized that whereas small luteal cells were described to react more robustly to LH stimulation and increase their P4 output strongly after stimulation, large luteal cells exhibit higher basal P4 production and less robust responsiveness to LH stimulation [61]. This could imply that the basal production of P4 in large CL and the constant provision of P4 remain under the control of HIF1α in large lutein cells (the involvement of HIF1α in these cells has been confirmed by Nishimura and Okuda, 2010, 2020 [5,45]). Furthermore, the localization of PHDs in the luteal cells appears to provide insights into their regulatory mechanisms in preventing exaggerated levels of HIF1α. Our results suggest that among the PHDs, both PHD2 and PHD3 are present, whereas PHD1 seemed to be less abundant in the luteal cells. Interestingly, PHD2 appeared to be ubiquitously distributed. The expression of PHDs has been shown to vary between different tissue types, as well as within the same tissue [13,62,63]. The relative importance of specific PHDs in regulating HIF1α in bovine luteal cells remains to be investigated. Additionally, VHL seems to contribute to the action of the PHDs, as it exhibited positive signals in both large and small luteal cells.

Another important ISH-based finding relates to the distribution of signals within the vascular bed. Here, we showed strong HIF1α signals being localized mainly in the capillaries, where the oxygen exchange and formation of new capillaries occurs. While angiogenesis takes place throughout the luteal lifespan, vascular endothelial growth factors, including VEGF, one of the best known downstream targets of HIF1α [64], are strongly expressed in the early luteal stage of the bovine CL, among other species [7,45]. Furthermore, the ISH results showed that PHD 1–3 were present in different cellular compartments of the luteal vessels. Their distribution was clearly compartmentalized, with PHD1 transcripts being predominant in the vascular bed, ranging from small capillaries to larger vessels, and less abundant in luteal cells. The other PHDs were distributed more ubiquitously. Silencing PHD2 has been shown in rat CL to result in increased HIF1α and subsequently VEGF [65]. The vascular presence of VHL in the luteal endothelial cells observed by ISH could encompass biological processes beyond angiogenesis control, such as cell adhesion, proliferation, immune response, and ROS prevention [66]. Interestingly, the accumulation of HIF1α in VHL-deficient endothelial tissue renders the tissue more fragile and increases its susceptibility to tumorigenesis, emphasizing the role of VHL in controlling HIF1α activity [66,67].

Cumulatively, this is the first time that HIF1α regulatory factors have been localized in the bovine CL, and their distinctive expression patterns indicate a cell-specific regulation of HIF1α availability. Overall, the spatial distribution of their respective transcripts within the bovine CL in the ISH study may be indicative of their role in the normal function of the gland.

The evaluation of CL biopsies during LPS challenge provided new insights into the possible involvement of HIF1α and oxygen-sensing factors in bovine luteal tissues. Although the corpus luteum (CL) is expected to be robust to injury, theoretically allowing for repeated biopsies, additional inflammatory insults were intentionally avoided to minimize potential effects. Consequently, we refrained from repeated measurements of the same CL before and after treatment. Instead, treatments were performed in consecutive cycles, and the cows served as their own controls. Studies in different human in vitro models, such as THC-1 myeloid leukaemia, macrophages, and epithelial ovarian cancer cell lines, demonstrated that LPS increases HIF1α transcription [68,69,70,71]. In the present study, HIF1α transcriptional expression in LPS-treated biopsies remained unaffected. This could be due to several factors, including the variability of the animals we evaluated, or the window of sample collection in the mid-luteal stage, where HIF1α is strongly represented and active. It could also reflect tissue-specific properties, with the bovine CL being able to stabilize HIF1α expression in response to external stressors. No significant differences were observed in *PHD2*, *FIH*, or *VHL* gene expression. However, LPS treatment affected the expression of *PHD1* and *PHD3*, which, taking into account the apparent predominance of PHD1 in vascular compartments, appears particularly interesting, as in vitro LPS treatment was shown to induce proinflammatory reactions in luteal endothelial cells, mirrored in increased *ICAM1* and *NFκB* expression [35]. These changes did not persist beyond the LPS-challenged reproductive cycle, indicating that the effects of LPS on the ovary may be transient and specific to the infection period, with no discernible impact on gene expression levels after recovery. It would be interesting to evaluate LPS-treated CL samples collected at early or late luteal stages to verify this hypothesis. Furthermore, the observed gene expression changes in the control groups might be considered physiologically normal, as such fluctuations are likely influenced by hormonal and dynamic luteal changes that occur during the oestrous cycle [72,73].

Apart from regulating HIF1α, PHDs have been proposed to be involved in the regulation of ICAM1 and NFκB [74,75,76,77]. *ICAM1* is involved in leukocyte recruitment and attachment to the endothelium, and induces vascular disruption and endothelial inflammation [39,78], while NFκB regulates immune and inflammatory responses [40]. Our results demonstrated a decrease in PHD1- and an increase in PHD3-encoding transcripts, while *NFKB2* and *ICAM1* expression were enhanced in the LPS-treated bovine CL. A previous study has shown that PHD3-deficient mice treated with LPS exhibited increased susceptibility to sepsis, increased pro-inflammatory cytokine release, enhanced macrophage activity, and elevated levels of NFκB and ICAM1 [74]. Another study demonstrated that PHD1 deficiency increased global NFκB expression in mouse hepatocytes [79]. Additionally, knockdown of PHD1 increased expression of NFκB in mice with myocardial ischemia/reperfusion injury [80]. Moreover, inhibition of PHD activity enhanced monocyte adherence, mediated by increased *ICAM1* expression in THP-1 human monocytes cocultured with EC52 rat endothelial cells [75]. The effects of LPS on *ICAM1* expression have also been observed in endothelial cells derived from human brain, umbilical vein, neonatal dermal lymphatics [81,82,83], and more recently in immortalized ovine luteal endothelial (OLENDO) cells [35]. The findings from our in vivo approach support the results obtained in vitro from the OLENDO cells model. The increased expression of *ICAM1* and *NFKB2* after LPS treatment further supports the role of PHDs in the immune response during luteal inflammation. Considering their spatial distribution as demonstrated by ISH, they might be involved in LPS-induced endothelial inflammation.

## 5. Conclusions

**Figure 5:**
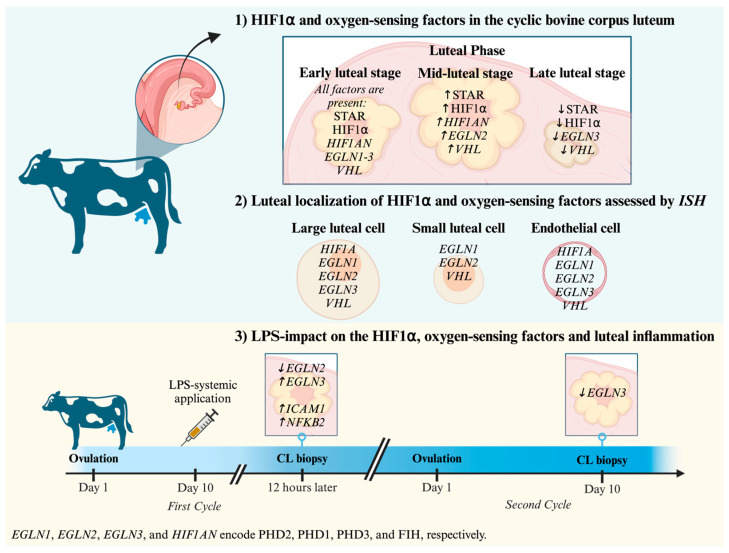
Schematic representation of the involvement of HIF1α and oxygen-sensing factors in the cyclic bovine corpus luteum, as well as their expression in the CL of cows treated with lipopolysaccharide (LPS; gram-negative bacterial endotoxin). The data regarding STAR derive from [32]. This figure was created with BioRender.com.

Our study provides new insights into the possible regulation of HIF1α in the bovine CL (summarized in the Figure 5), thereby introducing a new aspect to its functional implications in reproduction. While the mid-luteal stage appears to be a critical period characterized by increased HIF1α activity, our results suggest the involvement of PHDs, VHL, and FIH in regulating HIF1α levels throughout the CL lifespan. Furthermore, the changes observed in PHD expression in response to LPS treatment highlight the intricate interplay between lower oxygen tension and immune responses within the CL microenvironment, which may affect luteal function and progesterone production.

## Figures and Tables

**Table 1:** List of genes and corresponding TaqMan systems used for semi-quantitative real-time PCR.

Gene	Accession Numbers	Primer Sequence		Product Length (bp)
*HIF1A*	NM_174339.3	* Prod. Nr. Bt03259341_m1		109
*EGLN1* (PHD2)	NM_001206046.2	* Prod. Nr. Bt00991948_m1		105
*EGLN2* (PHD1)	NM_001102193.1	* Prod. Nr. Bt03273704_m1		87
*EGLN3* (PHD3)	NM_001101164.2	* Prod. Nr. Bt03272110_m1		67
*HIF1AN* (FIH)	NM_001083443.2	* Prod. Nr. Bt03256248_m1		75
*VHL*	NM_001110019.1	* Prod. Nr. Bt03276144_m1		103
*ICAM1*	NM_174348.2	* Prod. Nr. Bt03213906_m1		101
*NFKB2*	NM_001102101.1	* Prod. Nr. Bt03272792_g1		101
*ACTINB*	NM_173979.3	* Prod. Nr. Bt03279175_g1		144
*GAPDH*	NM_001034034	Forward	5′-GCGATACTCACTCTTCTACCTTCGA-3′	82
		Reverse	5′-TCGTACCAGGAAATGAGCTTGAC-3′	
		TaqMan probe	5′-CTGGCATTGCCCTCAACGACCACT-3′	

* Applied Biosystems product number(s).

**Table 2:** List of primers used for generating riboprobes for in situ hybridization (ISH).

Gene	Accession Numbers	Primer Sequences for ISH		Product Length (bp)
*EGLN1*	NM_001206046	Forward	5′-CTGGCGCTGGAGTACATC-3′	261
(PHD2)		Reverse	5′-CAGGTCGTCCATGTTGTTC-3′	
*EGLN2*	NM_001102193	Forward	5′-ACGGGCGCTGCATCACTTGT-3′	275
(PHD1)		Reverse	5′-CCCTTTCTGTCCTGATGCTA-3′	
*EGLN3*	NM_001101164	Forward	5′-GCTTGCTACCCAGGAAATG-3′	346
(PHD3)		Reverse	5′-CAGTAAGGGCAGGTTCAGTC-3′	
*VHL*	NM_001110019	Forward	5′-CCAGGTCATCTTCTGCAAC-3′	340
		Reverse	5′-GTAGAGGGATCGCACAATG-3′	
*HIF1A*	NM_174339.3	Forward	5′-CTCACCATCAGCTATTTGCG-3′	314
		Reverse	5′-CTCCGCTGTGTATTTTGCTC-3′	

## Data Availability

The original contributions presented in this study are included in the article. Further inquiries can be directed to the corresponding author.

## References

[1] Schams D., Berisha B. (2004). Regulation of Corpus Luteum Function in Cattle—An Overview. Reprod. Domest. Anim..

[2] Forde N., Beltman M.E., Lonergan P., Diskin M., Roche J.F., Crowe M.A. (2011). Oestrous Cycles in Bos Taurus Cattle. Anim. Reprod. Sci..

[3] Semenza G.L. (2000). HIF-1: Mediator of Physiological and Pathophysiological Responses to Hypoxia. J. Appl. Physiol..

[4] Semenza G.L. (1998). Hypoxia-Inducible Factor 1: Master Regulator of O_2_ Homeostasis. Curr. Opin. Genet. Dev..

[5] Nishimura R., Okuda K. (2020). Multiple Roles of Hypoxia in Bovine Corpus Luteum. J. Reprod. Dev..

[6] Kowalewski M.P., Gram A., Boos A. (2015). The Role of Hypoxia and HIF1α in the Regulation of STAR-Mediated Steroidogenesis in Granulosa Cells. Mol. Cell. Endocrinol..

[7] Meidan R., Klipper E., Zalman Y., Yalu R. (2013). The Role of Hypoxia-Induced Genes in Ovarian Angiogenesis. Reprod. Fertil. Dev..

[8] Juengel J.L., Garverick A., Johnson A.L., Youngquist R.S., Smith M.F. (1993). Apoptosis during Luteal Regression. Endocrinology.

[9] Nishimura R., Okuda K. (2015). Multiple Roles of Hypoxia in Ovarian Function: Roles of Hypoxia-Inducible Factor-Related and-Unrelated Signals during the Luteal Phase. Reprod. Fertil. Dev..

[10] Wise T.H., Caton D., Thatcher W.W., Barron D.H., Fields M.J. (1982). Ovarian Function during the Estrous Cycle of the Cow: Ovarian Blood Flow and Progesterone Release Rate. J. Anim. Sci..

[11] Nishimura R., Sakumoto R., Tatsukawa Y., Acosta T.J., Okuda K. (2006). Oxygen Concentration Is an Important Factor for Modulating Progesterone Synthesis in Bovine Corpus Luteum. Endocrinology.

[12] Guzy R.D., Schumacker P.T. (2006). Oxygen Sensing by Mitochondria at Complex III: The Paradox of Increased Reactive Oxygen Species during Hypoxia. Exp. Physiol..

[13] Gysin T., Kowalewski M.P. (2021). The Involvement of Hypoxia-Inducible Factor 1α (HIF1α)-Stabilising Factors in Steroidogenic Acute Regulatory (STAR) Protein-Dependent Steroidogenesis in Murine KK1 Granulosa Cells in Vitro. Reprod. Fertil. Dev..

[14] Wiener C.M., Booth G., Semenza G.L. (1996). In Vivo Expression of MRNAs Encoding Hypoxia-Inducible Factor 1. Biochem. Biophys. Res. Commun..

[15] Wang G.L., Jiang B.H., Rue E.A., Semenza G.L. (1995). Hypoxia-Inducible Factor 1 Is a Basic-Helix-Loop-Helix-PAS Heterodimer Regulated by Cellular O2 Tension. Proc. Natl. Acad. Sci. USA.

[16] Alam H., Maizels E.T., Park Y., Ghaey S., Feiger Z.J., Chandel N.S., Hunzicker-Dunn M. (2004). Follicle-Stimulating Hormone Activation of Hypoxia-Inducible Factor-1 by the Phosphatidylinositol 3-Kinase/AKT/Ras Homolog Enriched in Brain (Rheb)/Mammalian Target of Rapamycin (MTOR) Pathway Is Necessary for Induction of Select Protein Markers of Follic. J. Biol. Chem..

[17] Fukuda R., Hirota K., Fan F., Jung Y.D., Ellis L.M., Semenza G.L. (2002). Insulin-like Growth Factor 1 Induces Hypoxia-Inducible Factor 1-Mediated Vascular Endothelial Growth Factor Expression, Which Is Dependent on MAP Kinase and Phosphatidylinositol 3-Kinase Signaling in Colon Cancer Cells. J. Biol. Chem..

[18] Rico C., Dodelet-Devillers A., Paquet M., Tsoi M., Lapointe E., Carmeliet P., Boerboom D. (2014). HIF1 Activity in Granulosa Cells Is Required for FSH-Regulated Vegfa Expression and Follicle Survival in Mice. Biol. Reprod..

[19] Huang L.E., Arany Z., Livingston D.M., Franklin Bunn H. (1996). Activation of Hypoxia-Inducible Transcription Factor Depends Primarily upon Redox-Sensitive Stabilization of Its α Subunit. J. Biol. Chem..

[20] Bruick R.K., McKnight S.L. (2001). A Conserved Family of Prolyl-4-Hydroxylases That Modify HIF. Science.

[21] Epstein A.C.R., Gleadle J.M., McNeill L.A., Hewitson K.S., O’Rourke J., Mole D.R., Mukherji M., Metzen E., Wilson M.I., Dhanda A. (2001). *C. Elegans* EGL-9 and Mammalian Homologs Define a Family of Dioxygenases That Regulate HIF by Prolyl Hydroxylation. Cell.

[22] Elkins J.M., Hewitson K.S., McNeill L.A., Seibel J.F., Schlemminger I., Pugh C.W., Ratcliffe P.J., Schofield C.J. (2003). Structure of Factor-Inhibiting Hypoxia-Inducible Factor (HIF) Reveals Mechanism of Oxidative Modification of HIF-1α. J. Biol. Chem..

[23] Hewitson K.S., McNeill L.A., Riordan M.V., Tian Y.M., Bullock A.N., Welford R.W., Elkins J.M., Oldham N.J., Bhattacharya S., Gleadle J.M. (2002). Hypoxia-Inducible Factor (HIF) Asparagine Hydroxylase Is Identical to Factor Inhibiting HIF (FIH) and Is Related to the Cupin Structural Family. J. Biol. Chem..

[24] Freedman S.J., Sun Z.Y.J., Poy F., Kung A.L., Livingston D.M., Wagner G., Eck M.J. (2002). Structural Basis for Recruitment of CBP/P300 by Hypoxia-Inducible Factor-1α. Proc. Natl. Acad. Sci. USA.

[25] Jaakkola P., Mole D.R., Tian Y.M., Wilson M.I., Gielbert J., Gaskell S.J., Von Kriegsheim A., Hebestreit H.F., Mukherji M., Schofield C.J. (2001). Targeting of HIF-α to the von Hippel-Lindau Ubiquitylation Complex by O2-Regulated Prolyl Hydroxylation. Science.

[26] Huang L.E., Gu J., Schau M., Bunn H.F. (1998). Regulation of Hypoxia-Inducible Factor 1α Is Mediated by an O2-Dependent Degradation Domain via the Ubiquitin-Proteasome Pathway. Proc. Natl. Acad. Sci. USA.

[27] Hertl J.A., Gröhn Y.T., Leach J.D.G., Bar D., Bennett G.J., González R.N., Rauch B.J., Welcome F.L., Tauer L.W., Schukken Y.H. (2010). Effects of Clinical Mastitis Caused by Gram-Positive and Gram-Negative Bacteria and Other Organisms on the Probability of Conception in New York State Holstein Dairy Cows. J. Dairy Sci..

[28] Sheldon I.M., Williams E.J., Miller A.N.A., Nash D.M., Herath S. (2008). Uterine Diseases in Cattle after Parturition. Vet. J..

[29] Sheldon I.M., Rycroft A.N., Dogan B., Craven M., Bromfield J.J., Chandler A., Roberts M.H., Price S.B., Gilbert R.O., Simpson K.W. (2010). Specific Strains of Escherichia Coli Are Pathogenic for the Endometrium of Cattle and Cause Pelvic Inflammatory Disease in Cattle and Mice. PLoS ONE.

[30] Barker A.R., Schrick F.N., Lewis M.J., Dowlen H.H., Oliver S.P. (1998). Influence of Clinical Mastitis during Early Lactation on Reproductive Performance of Jersey Cows. J. Dairy Sci..

[31] Sheldon I.M., Cronin J., Goetze L., Donofrio G., Schuberth H.J. (2009). Defining Postpartum Uterine Disease and the Mechanisms of Infection and Immunity in the Female Reproductive Tract in Cattle. Biol. Reprod..

[32] Lüttgenau J., Herzog K., Strüve K., Latter S., Boos A., Bruckmaier R.M., Bollwein H., Kowalewski M.P. (2016). LPS-Mediated Effects and Spatio-Temporal Expression of TLR2 and TLR4 in the Bovine Corpus Luteum. Reproduction.

[33] Herzog K., Strüve K., Kastelic J.P., Piechotta M., Ulbrich S.E., Pfarrer C., Shirasuna K., Shimizu T., Miyamoto A., Bollwein H. (2012). Escherichia Coli Lipopolysaccharide Administration Transiently Suppresses Luteal Structure and Function in Diestrous Cows. Reproduction.

[34] Qu X., Yan L., Guo R., Li H., Shi Z. (2019). ROS-Induced GATA4 and GATA6 Downregulation Inhibits StAR Expression in LPS-Treated Porcine Granulosa-Lutein Cells. Oxid. Med. Cell. Longev..

[35] Gram A., Kowalewski M.P. (2022). Molecular Mechanisms of Lipopolysaccharide (LPS) Induced Inflammation in an Immortalized Ovine Luteal Endothelial Cell Line (OLENDO). Vet. Sci..

[36] Gram A., Grazul-Bilska A.T., Boos A., Rahman N.A., Kowalewski M.P. (2019). Lipopolysaccharide Disrupts Gap Junctional Intercellular Communication in an Immortalized Ovine Luteal Endothelial Cell Line. Toxicol. Vitr..

[37] Hortelano S., López-Fontal R., Través P.G., Villa N., Grashoff C., Boscá L., Luque A. (2010). ILK Mediates LPS-Induced Vascular Adhesion Receptor Expression and Subsequent Leucocyte Trans-Endothelial Migration. Cardiovasc. Res..

[38] Takeuchi S., Kawashima S., Rikitake Y., Ueyama T., Inoue N., Hirata K.I., Yokoyama M. (2000). Cerivastatin Suppresses Lipopolysaccharide-Induced ICAM-1 Expression through Inhibition of Rho GTPase in BAEC. Biochem. Biophys. Res. Commun..

[39] Dragoni S., Hudson N., Kenny B.-A., Burgoyne T., McKenzie J.A., Gill Y., Blaber R., Futter C.E., Adamson P., Greenwood J. (2017). Endothelial MAPKs Direct ICAM-1 Signaling to Divergent Inflammatory Functions. J. Immunol..

[40] Vallabhapurapu S., Karin M. (2009). Regulation and Function of NF-ΚB Transcription Factors in the Immune System. Annu. Rev. Immunol..

[41] Miyamoto Y., Skarzynski D.J., Okuda K. (2000). Is Tumor Necrosis Factor a Trigger for the Initiation of Endometrial Prostaglandin F(2α) Release at Luteolysis in Cattle?. Biol. Reprod..

[42] Xie F., Wang J., Zhang B. (2023). RefFinder: A Web-Based Tool for Comprehensively Analyzing and Identifying Reference Genes. Funct. Integr. Genomics.

[43] Kowalewski M.P., Mason J.I., Howie A.F., Morley S.D., Schuler G., Hoffmann B. (2006). Characterization of the Canine 3β-Hydroxysteroid Dehydrogenase and Its Expression in the Corpus Luteum during Diestrus. J. Steroid Biochem. Mol. Biol..

[44] Berisha B., Schams D., Rodler D., Pfaffl M.W. (2015). Angiogenesis in The Ovary—The Most Important Regulatory Event for Follicle and Corpus Luteum Development and Function in Cow—An Overview. J. Vet. Med. Ser. C Anat. Histol. Embryol..

[45] Nishimura R., Okuda K. (2010). Hypoxia Is Important for Establishing Vascularization during Corpus Luteum Formation in Cattle. J. Reprod. Dev..

[46] Hasegawa H., Nishimura R., Yamashita M., Yamaguchi T., Hishinuma M., Okuda K. (2019). Effect of Hypoxia on Progesterone Production by Cultured Bovine Early and Mid Luteal Cells. J. Reprod. Dev..

[47] Baddela V.S., Sharma A., Michaelis M., Vanselow J. (2020). HIF1 Driven Transcriptional Activity Regulates Steroidogenesis and Proliferation of Bovine Granulosa Cells. Sci. Rep..

[48] Klipper E., Levit A., Mastich Y., Berisha B., Schams D., Meidan R. (2010). Induction of Endothelin-2 Expression by Luteinizing Hormone and Hypoxia: Possible Role in Bovine Corpus Luteum Formation. Endocrinology.

[49] Sousa L.M.M.D.C., Silva R.d.S., da Fonseca V.U., Leandro R.M., Di Vincenzo T.S., Alves-Wagner A.B., Machado U.F., De Papa P.C. (2016). Is the Canine Corpus Luteum an Insulin-Sensitive Tissue?. J. Endocrinol..

[50] Kumar A., Rani L., Dhole B. (2014). Role of Oxygen in the Regulation of Leydig Tumor Derived MA-10 Cell Steroid Production: The Effect of Cobalt Chloride. Syst. Biol. Reprod. Med..

[51] Fadhillah, Yoshioka S., Nishimura R., Yamamoto Y., Kimura K., Okuda K. (2017). Hypoxia-Inducible Factor 1 Mediates Hypoxia-Enhanced Synthesis of Progesterone during Luteinization of Granulosa Cells. J. Reprod. Dev..

[52] Miikkulainen P., Högel H., Rantanen K., Suomi T., Kouvonen P., Elo L.L., Jaakkola P.M. (2017). HIF Prolyl Hydroxylase PHD3 Regulates Translational Machinery and Glucose Metabolism in Clear Cell Renal Cell Carcinoma. Cancer Metab..

[53] Yano H., Sakai M., Matsukawa T., Yagi T., Naganuma T., Mitsushima M., Iida S., Inaba Y., Inoue H., Unoki-Kubota H. (2018). PHD3 Regulates Glucose Metabolism by Suppressing Stress-Induced Signalling and Optimising Gluconeogenesis and Insulin Signalling in Hepatocytes. Sci. Rep..

[54] Dupont J., Scaramuzzi R.J. (2016). Insulin Signalling and Glucose Transport in the Ovary and Ovarian Function during the Ovarian Cycle. Biochem. J..

[55] Nishimoto H., Matsutani R., Yamamoto S., Takahashi T., Hayashi K.G., Miyamoto A., Hamano S., Tetsuka M. (2006). Gene Expression of Glucose Transporter (GLUT) 1, 3 and 4 in Bovine Follicle and Corpus Luteum. J. Endocrinol..

[56] Chen C., Pore N., Behrooz A., Ismail-Beigi F., Maity A. (2001). Regulation of Glut1 MRNA by Hypoxia-Inducible Factor-1: Interaction between H-Ras and Hypoxia. J. Biol. Chem..

[57] Nishimura R., Hasegawa H., Yamashita M., Ito N., Okamoto Y., Takeuchi T., Kubo T., Iga K., Kimura K., Hishinuma M. (2017). Hypoxia Increases Glucose Transporter 1 Expression in Bovine Corpus Luteum at the Early Luteal Stage. J. Vet. Med. Sci..

[58] Chase C.C., Del Vecchio R.P., Smith S.B., Randel R.D. (1992). In Vitro Metabolism of Glucose by Bovine Reproductive Tissues Obtained during the Estrous Cycle and after Calving. J. Anim. Sci..

[59] Wang X., Zou Z., Yang Z., Jiang S., Lu Y., Wang D., Dong Z., Xu S., Zhu L. (2019). HIF 1 Inhibits STAR Transcription and Testosterone Synthesis in Murine Leydig Cells. J. Mol. Endocrinol..

[60] Berisha B., Schams D., Rodler D., Sinowatz F., Pfaffl M.W. (2017). Expression Pattern of HIF1alpha and Vasohibins during Follicle Maturation and Corpus Luteum Function in the Bovine Ovary. Reprod. Domest. Anim..

[61] Weber D.M., Fields P.A., Romrell L.J., Tumwasorn S., Ball B.A., Drost M., Fields M.J. (1987). Functional Differences between Small and Large Luteal of the Late-Pregnant vs. Nonpregnant Cow. Biol. Reprod..

[62] Soilleux E.J., Turley H., Tian Y.M., Pugh C.W., Gatter K.C., Harris A.L. (2005). Use of Novel Monoclonal Antibodies to Determine the Expression and Distribution of the Hypoxia Regulatory Factors PHD-1, PHD-2, PHD-3 and FIH in Normal and Neoplastic Human Tissues. Histopathology.

[63] Fong G.H., Takeda K. (2008). Role and Regulation of Prolyl Hydroxylase Domain Proteins. Cell Death Differ..

[64] Semenza G.L. (2003). Targeting HIF-1 for Cancer Therapy. Nat. Rev. Cancer.

[65] Zhang Z., Tang Z., Wang Z., Pang X., Yin D. Effects of HIF Prolyl Hydoxylase-2 Silencing on Hypoxia-Induced Vascular Endothelial Growth Factor Expression in Luteal Cells. Proceedings of the International Conference on Engineering Technologies, Engineering Education and Engineering Management (ETEEEM 2014).

[66] de Rojas-P I., Albiñana V., Taranets L., Recio-Poveda L., Cuesta A.M., Popov N., Kronenberger T., Botella L.M. (2021). The Endothelial Landscape and Its Role in von Hippel–Lindau Disease. Cells.

[67] Singh A.D., Shields C.L., Shields J.A. (2001). Von Hippel-Lindau Disease. Surv. Ophthalmol..

[68] Blouin C.C., Pagé E.L., Soucy G.M., Richard D.E. (2004). Hypoxic Gene Activation by Lipopolysaccharide in Macrophages: Implication of Hypoxia-Inducible Factor 1α. Blood.

[69] Chen Q., Cui K., Zhao Z., Xu X., Liu Y., Shen Y., Chen F., Mai K., Ai Q. (2022). LPS Stimulation Stabilizes HIF-1α by Enhancing HIF-1α Acetylation via the PARP1-SIRT1 and ACLY-Tip60 Pathways in Macrophages. FASEB J..

[70] Nishi K., Oda T., Takabuchi S., Oda S., Fukuda K., Adachi T., Semenza G.L., Shingu K., Hirota K. (2008). LPS Induces Hypoxia-Inducible Factor 1 Activation in Macrophage- Differentiated Cells in a Reactive Oxygen Species-Dependent Manner. Antioxidants Redox Signal..

[71] Zhao B., Niu X., Huang S., Yang J., Wei Y., Wang X., Wang J., Wang Y., Guo X. (2022). TLR4 Agonist and Hypoxia Synergistically Promote the Formation of TLR4/NF- κ B/HIF-1 α Loop in Human Epithelial Ovarian Cancer. Anal. Cell. Pathol..

[72] Kowalik M.K., Dobrzyn K., Mlynarczuk J., Rekawiecki R. (2022). Effect of Steroid Hormones, Prostaglandins (E2 and F2α), Oxytocin, and Tumor Necrosis Factor Alpha on Membrane Progesterone (P4) Receptors Gene Expression in Bovine Myometrial Cells. Animals.

[73] Rekawiecki R., Kowalik M.K., Kotwica J. (2017). The Expression of Progesterone Receptor Coregulators MRNA and Protein in Corpus Luteum and Endometrium of Cows during the Estrous Cycle. Anim. Reprod. Sci..

[74] Kiss J., Mollenhauer M., Walmsley S.R., Kirchberg J., Radhakrishnan P., Niemietz T., Dudda J., Steinert G., Whyte M.K.B., Carmeliet P. (2012). Loss of the Oxygen Sensor PHD3 Enhances the Innate Immune Response to Abdominal Sepsis. J. Immunol..

[75] Winning S., Splettstoesser F., Fandrey J., Frede S. (2010). Acute Hypoxia Induces HIF-Independent Monocyte Adhesion to Endothelial Cells through Increased Intercellular Adhesion Molecule-1 Expression: The Role of Hypoxic Inhibition of Prolyl Hydroxylase Activity for the Induction of NF-ΚB. J. Immunol..

[76] Takeda K., Ichiki T., Narabayashi E., Inanaga K., Miyazaki R., Hashimoto T., Matsuura H., Ikeda J., Miyata T., Sunagawa K. (2009). Inhibition of Prolyl Hydroxylase Domain-Containing Protein Suppressed Lipopolysaccharide-Induced TNF-α Expression. Arterioscler. Thromb. Vasc. Biol..

[77] Cummins E.P., Seeballuck F., Keely S.J., Mangan N.E., Callanan J.J., Fallon P.G., Taylor C.T. (2008). The Hydroxylase Inhibitor Dimethyloxalylglycine Is Protective in a Murine Model of Colitis. Gastroenterology.

[78] Morise Z., Eppihimer M., Granger D.N., Anderson D.C., Grisham M.B. (1999). Effects of Lipopolysaccharide on Endothelial Cell Adhesion Molecule Expression in Interleukin-10 Deficient Mice. Inflammation.

[79] Fitzpatrick S.F., Fábián Z., Schaible B., Lenihan C.R., Schwarzl T., Rodriguez J., Zheng X., Li Z., Tambuwala M.M., Higgins D.G. (2016). Prolyl Hydroxylase-1 Regulates Hepatocyte Apoptosis in an NF-ΚB-Dependent Manner. Biochem. Biophys. Res. Commun..

[80] Adluri R.S., Thirunavukkarasu M., Dunna N.R., Zhan L., Oriowo B., Takeda K., Sanchez J.A., Otani H., Maulik G., Fong G.H. (2011). Disruption of Hypoxia-Inducible Transcription Factor-Prolyl Hydroxylase Domain-1 (PHD-1 -/-) Attenuates Ex Vivo Myocardial Ischemia/Reperfusion Injury through Hypoxia-Inducible Factor-1α Transcription Factor and Its Target Genes in Mice. Antioxidants Redox Signal..

[81] Sawa Y., Ueki T., Hata M., Iwasawa K., Tsuruga E., Kojima H., Ishikawa H., Yoshida S. (2008). LPS-Induced IL-6, IL-8, VCAM-1, and ICAM-1 Expression in Human Lymphatic Endothelium. J. Histochem. Cytochem..

[82] Wong D., Dorovini-Zis K. (1992). Upregulation of Intercellular Adhesion Molecule-1 (ICAM-1) Expression in Primary Cultures of Human Brain Microvessel Endothelial Cells by Cytokines and Lipopolysaccharide. J. Neuroimmunol..

[83] Yan W., Zhao K., Jiang Y., Huang Q., Wang J., Kan W., Wang S. (2002). Role of P38 MAPK in ICAM-1 Expression of Vascular Endothelial Cells Induced by Lipopolysaccharide. Shock.

